# Effects of Low-Intensity Aaerobic Training on Cardiac Dysfunction and Myocardial Fibrosis Induced by Doxorubicin in Wistar Rats

**DOI:** 10.1007/s12012-026-10150-7

**Published:** 2026-06-30

**Authors:** Talita Cristina Rodrigues Pereira, Alinne Tatiane Faria Silva, Matheus Matioli Mantovani, Simone Ramos Deconte, Bruno Antônio Ferreira, Mariane Marques da Guarda Pinto, Paulo Ricardo Lopes, Taysa Machado Menezes, Thiago Montes Fidale, Yara Cristina de Paiva Maia, Elmiro Santos Resende

**Affiliations:** 1 Experimental Medicine Laboratory, Universidade Federal de Uberlândia-UFU, Umuarama Campus - Block UMU2H, Room 01 Ave. Pará - 1720 – Neighborhood Umuarama, Uberlândia, MG Brazil; 2 Palliative Care and Health-Related Quality of Life Research Group, Barretos Cancer Hospital, Barretos, SP Brazil; 3 Universidade Federal de Uberlândia-UFU, Uberlândia, MG Brazil; 4 Laboratory Technician/Biology, Institute of Biomedical Sciences, Department of Physiology, Universidade Federal de Uberlândia-UFU, Uberlândia, MG Brazil; 5 Institute of Biomedical Sciences of the Universidade Federal de Uberlândia - UFU, Universidade Federal do ABC, Uberlândia, Brazil; 6 Center for Natural and Human Sciences, Universidade Federal do ABC, Santo André, SP Brazil; 7 Universidade Federal de Uberlândia- Bioterium Network (REBIR-UFU), Uberlândia, MG Brazil; 8 Clinical Analysis Laboratory Technician ULAC-HC-UFU, Uberlândia, MG Brazil; 9 Barretos Cancer Hospital, Barretos, SP Brazil; 10 Department of Medicine, Universidade Federal de Catalão - UFCAT, Catalão, Goiás Brazil; 11 Postgraduate Program in Health Sciences, Postgraduate Program in Genetics and Biochemistry, Universidade Federal de Uberlândia-UFU, Uberlândia, MG Brazil; 12 Postgraduate Program in Health Sciences - PPGCS, Faculty of Medicine, Universidade Federal de Uberlândia- UFU, Uberlândia, MG Brazil

**Keywords:** Experimental model, Cardiovascular adaptation, Fibrosis, Chronic exercise training

## Abstract

Doxorubicin-induced cardiotoxicity is a major limitation in cancer therapy. Physical exercise has been proposed as a non-pharmacological strategy capable of mitigating these effects through cardiovascular adaptations. This study evaluated whether low-intensity aerobic training attenuates cardiac dysfunction induced by doxorubicin. Twenty-five adult male Wistar rats (450 and 500 g) were allocated and divided into four groups: Control (C), Doxorubicin (D), Doxorubicin and Trained (DT), and Trained (T). The D and DT groups received weekly intraperitoneal injections of doxorubicin over a four-week period, totaling a cumulative dose of 8 mg/kg. The DT and T groups underwent low-intensity aerobic training for 25 weeks, three times a week, with sessions lasting 10 to 20 min at 50 and 60% of maximum aerobic speed. Statistical analysis was performed using GraphPad Prism software (version 8.0). The D group showed a significant increase in myocardial collagen deposition and a reduction in S wave values (parietal and septal) on echocardiography, compared to the control group. The values observed were 0.040 for group D and 0.065 for group C in the parietal S wave (overall *p* = 0.0248), and 0.040 for group D and 0.070 for group C in the septal S wave (overall *p* = 0.0389). Regarding exercise tolerance, group D showed the greatest reduction in performance from Test 0 to Test 5 (65.06%; *p* < 0.0001), followed by group C (23.60%; *p* = 0.0103). Group DT showed a smaller numerical reduction in performance (23.07%), but this change did not reach statistical significance (*p* = 0.1256), while group T showed a non-significant numerical improvement (10.99%; *p* = 0.2020). The results suggest that low-intensity aerobic training may attenuate cardiac fibrosis and partially preserve functional capacity in rats with doxorubicin-induced cardiotoxicity.

**Graphical Abstract:**

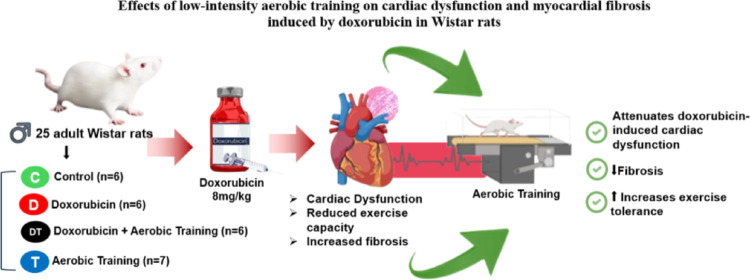

Doxorubicin-induced cardiotoxicity increases myocardial fibrosis and promotes cardiac remodeling compatible with cardiac dysfunction. In this study, low-intensity aerobic exercise appeared to attenuate collagen accumulation and partially preserve functional parameters in Wistar rats exposed to doxorubicin. Echocardiographic and histological analyses revealed preserved systolic function and reduced deposition of type I and III collagen in animals that received doxorubicin and underwent training. These findings suggest that low-intensity aerobic training may act as a protective strategy, by mitigating myocardial remodeling and contributing to better tolerance to doxorubicin-induced injury.

**Supplementary Information:**

The online version contains supplementary material available at 10.1007/s12012-026-10150-7.

## Introduction

Heart failure is one of the leading causes of global morbidity and mortality [1], characterized by deterioration of ventricular structure and function, resulting in reduced cardiac filling and/or ejection capacity. Among the factors contributing to its development, chemotherapy-induced cardiotoxicity has emerged as a major concern [2]. Doxorubicin, an anthracycline widely used in cancer treatment, is highly effective due to its multiple mechanisms of action, including topoisomerase II inhibition, free radical generation, and DNA intercalation [3, 4].

Despite its therapeutic efficacy, doxorubicin causes significant cardiotoxic effects. One of the primary mechanisms involves the inhibition of topoisomerase IIβ, resulting in double-strand DNA breaks that impair DNA repair and replication, as well as repress the transcription of genes essential for mitochondrial biogenesis and energy metabolism [5]. Concomitantly, doxorubicin increases oxidative stress, triggering lipid peroxidation [6], weakening mitochondrial membrane potential, allowing the mitochondrial permeability transition process, thus promoting mitochondrial dysfunction [7], compromising ATP, production and activating intrinsic apoptotic pathways involving cytochrome c release and caspase activation [8, 9]. These processes contribute to progressive myocardial injury, culminating in cardiac remodeling and necrosis.

Although doxorubicin improves cancer survival, its chronic cardiotoxicity compromises functional capacity and quality of life [10]. In this context, it is essential to identify effective adjuvant strategies to mitigate these adverse cardiac effects, which are characterized by ventricular wall dilation and thinning [11–13]. Thus, techniques such as tissue Doppler imaging (TDI) in echocardiography enable the detection of early systolic and diastolic abnormalities that may occur in heart failure-like cardiotoxic phenotypes [14, 15]. Furthermore, cardiac fibrosis represents a fundamental pathological feature, being directly related to the amount of collagen fibers present in the extracellular matrix, and is an important pathological factor in cardiac remodeling [16]. Both markers allow the detection of cardiomyopathy induced by the administration of doxorubicin.

Physical inactivity is associated with nearly a twofold increase in the risk of mortality, while light exercise may be linked to better clinical outcomes [17]. Aerobic exercise, in particular, is effective in reducing low-density lipoprotein (LDL) oxidation and inflammation levels, factors that are directly related to cardiovascular health protection and cardiovascular risk prevention [18].

In this context, aerobic training has been proposed as a promising non-pharmacological strategy capable of mitigating doxorubicin-induced cardiotoxicity [19–21]. Experimental studies have shown that exercise can attenuate cardiac injury by improving ventricular function, increasing myocardial compliance, and mitigating fibrotic remodeling, thereby promoting various beneficial adaptations [22].

Given this, the present study aimed to evaluate whether low-intensity aerobic exercise can attenuate the harmful effects of doxorubicin in Wistar rats, focusing on cardiac function, myocardial fibrosis, and exercise tolerance. The study hypothesis is that training is capable of mitigating the development of cardiotoxicity and preserving the functional capacity of these animals.

## Materials and Methods

### Ethical Approval

Twenty-five adult male Wistar rats with a mean age of 27 weeks were used in this study. At baseline, body weight ranged from 450 to 500 g, and at the end of the experiment, from 480 to 560 g. The animals were obtained from and housed at the Central Animal Facility Network of the Federal University of Uberlândia (REBIR-UFU) under controlled environmental conditions (22 ± 2 °C, relative humidity 40–60%, a 12-hour light/dark cycle, and adequate air exchange).

Housing was provided in polypropylene mini-isolators measuring 26.4 × 34.1 × 50.1 cm, with a total floor area of 1,154 cm², lined with flaked wood shavings, which were replaced weekly. Stocking density was determined based on the animals’ weight, with a maximum of 2 individuals per cage. Water dispensers were replaced once a week, in accordance with the REBIR-UFU protocol. Maintenance ration and water were provided *ad libitum*.

Throughout the study, feed intake was assessed indirectly through daily observation of the animals’ general condition, including feeding behavior, as well as monitoring of body weight and overall clinical condition, and the availability of feed and water in the containers. All experimental procedures were performed at the REBIR-UFU facilities, in accordance with the ethical protocol of the Animal Experimentation Ethics Committee (CEUA), and were approved under CEUA registration number 23117.04515/2022-71.

### Experimental Protocol

The animals were divided into four groups: the group treated with doxorubicin (Group D; *n* = 6), the group treated with doxorubicin and subjected to aerobic training (Group DT; *n* = 6), the group treated with saline (Group C; *n* = 6), and the group treated with saline and subjected to aerobic training (Group T; *n* = 7). The initial allocation of animals was based on the order in which they reached the minimum body weight required for the experiment. As the animals met this criterion, they were assigned alternately to groups D, DT, C, and T until both were complete.

**Fig. 1:**
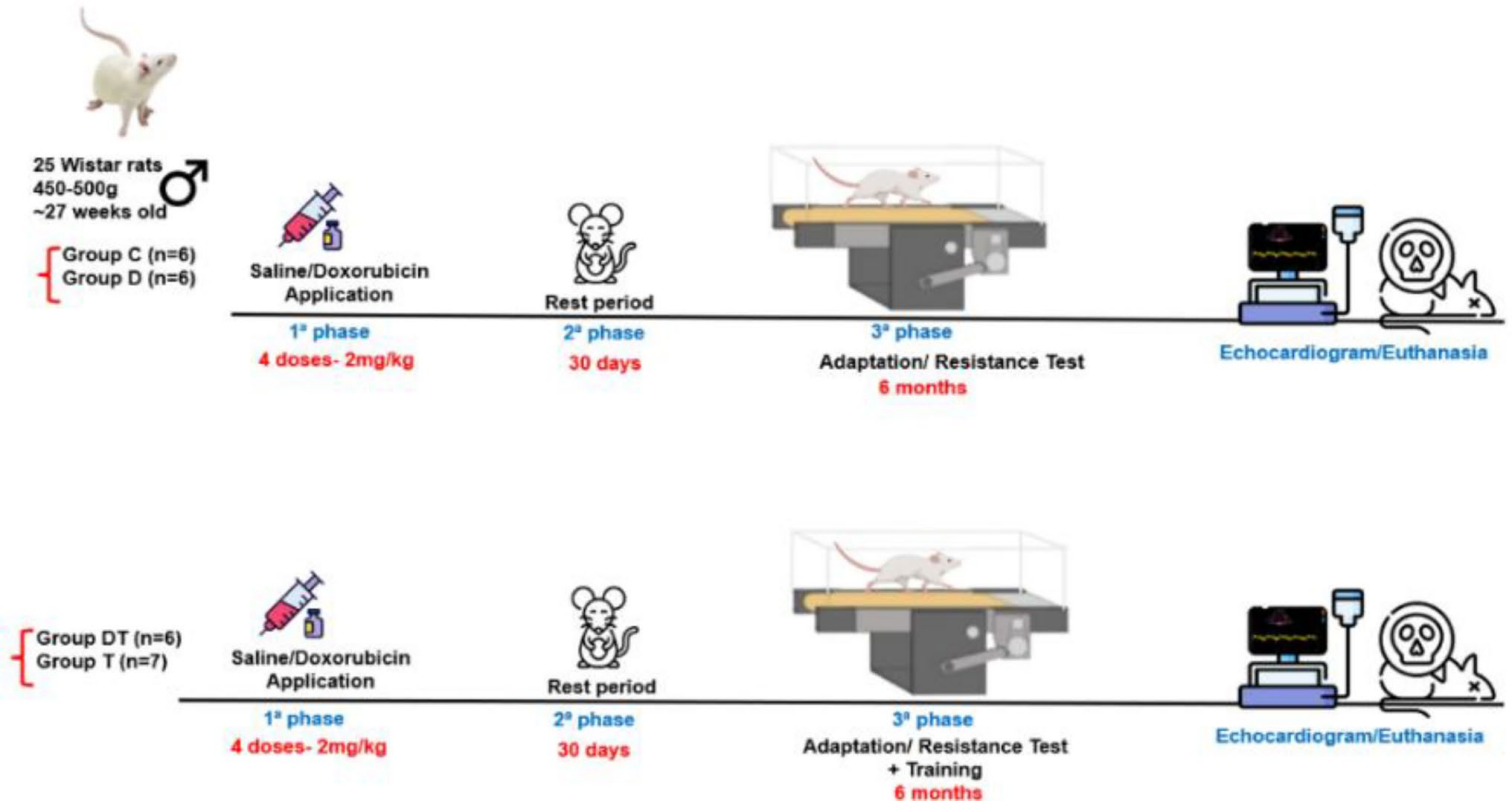
Experimental design and study timeline

The experimental protocol consisted of three phases. In the first phase, groups D and DT received doxorubicin, and groups C and T received saline solution. All groups had a one-month rest period after drug administration so that cardiotoxicity could develop in the animals treated with the drug, corresponding to the second phase. In the third phase, the animals in groups DT and T began the training protocol, which lasted approximately six months. An echocardiogram was then performed before euthanasia, as shown in (Fig. 1).

### Doxorubicin Application Protocol

All animals in groups D and DT received intraperitoneal injections of doxorubicin 2 mg/kg once a week for four weeks, reaching a cumulative dose of 8 mg/kg, reproducing a chronic model of doxorubicin-induced cardiotoxicity associated with cardiac dysfunction and myocardial remodeling in rats [23]. Groups C and T received similar doses of saline solution, using the same regimen as for the animals treated with doxorubicin. All injections were prepared under sterile conditions immediately before administration.

### Acclimatization on the Treadmill

All animals underwent a two-week acclimatization period on the EP 131 motorized treadmill for rats (Insight, Brazil), twice a week, with a training volume of 10 min, at an intensity of 10 m/min and an incline of 10° [24].

### Stress Test

Load increment tests were performed on a treadmill to assess the animals’ physical conditioning through their physical endurance over time, at different moments: before starting training, and at the end of each training cycle, which consisted of five weeks, in order to provide the animals with adequate training overload and aerobic training.

**Fig. 2:**
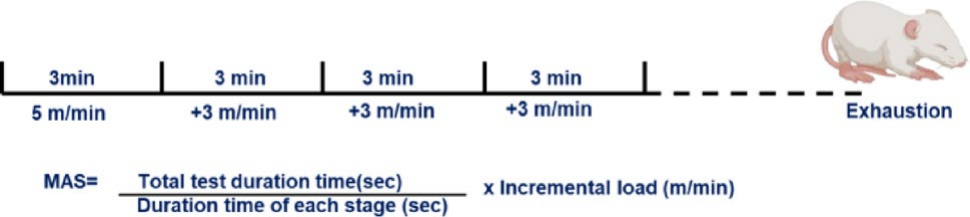
Incremental treadmill stress test protocol for determination of maximum aerobic speed (MAS)

The incremental load test as a function of time was adapted for the treadmill. The test consisted of 3-minute stages with an initial load of 5 m/minute. After the end of the third minute of each stage, a load of 3 m/minute was added to the treadmill speed until the animal was exhausted [25]. Exhaustion was determined by observing the following situations: (i) when the animal stopped running for more than 10 s or (ii) when it touched the back of the bay for the third time in the same stage after failing to pass the previous half of the bay in the same stage. After the tests, we obtained the Maximum aerobic speed (MAS) of each animal, as shown in (Fig. 2).

MAS was based on the average performance of the group to calculate the training speed, corresponding to 50% and 60% of the MAS achieved by the animals, in order not to overload the animals with speeds well above what they could handle. Only animals with performance within the same stage of the test were trained at the same time.

### Training Protocol

**Fig. 3:**
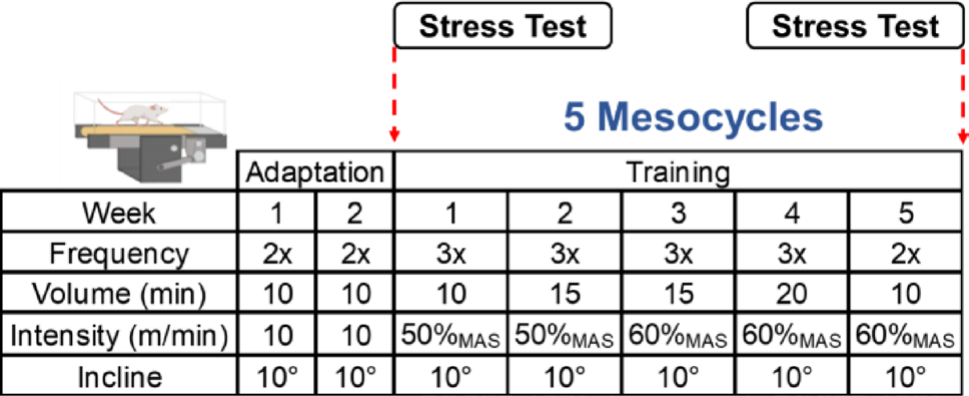
Aerobic Adaptation/Low-intensity aerobic training protocol

The training was adapted [26]. For the DT and T groups, treadmill running was implemented three times a week for five weeks (microcycles). The training volume was 10 min in the first week, 15 min in the second and third weeks, and 20 min in the fourth week of training. The treadmill speed started at 50% of the maximum speed obtained during a maximum effort test on a treadmill, with an incline of 10° (for two weeks) and was then increased to 60% (for the following three weeks). After the fourth week of training, regenerative training was performed (Taper - light training - fifth week), maintaining the intensity at 60%, a volume of 10 min, a frequency of twice a week, and a treadmill incline of 10° to enhance the physiological adaptations caused by training [27], totaling 5 training mesocycles as shown in (Fig. 3).

### Animal Monitoring and Humane Endpoints

From the start of the experimental protocol, all animals received environmental enrichment in accordance with REBIR-UFU guidelines. The animals were monitored daily through clinical evaluations and behavioral observations. The criteria established for defining the humane endpoint were based on the identification of consistent clinical signs of pain or impaired well-being. These included, among others, weight loss exceeding 15–20% of baseline, apathy, difficulty moving, or inability to maintain a natural posture, as well as respiratory changes such as dyspnea, tachypnea, or irregular breathing patterns. The presence of any of these signs was interpreted as indicative of significant suffering. Pain assessment was supplemented by the systematic application of the Rat Grimace Scale, performed in a standardized manner, with moderate to high scores interpreted as indicative of significant pain.

Although humane euthanasia criteria were established and animals were monitored daily throughout the experimental period, no animal met the criteria for early euthanasia. Therefore, all animals completed the experimental protocol and were euthanized only at the end of the experiment.

### Echocardiographic Analysis

All animals were placed in lateral recumbency by manual restraint and anesthetized with isoflurane at a rate of 4% and maintained for a short period at 2% [28, 29] to perform echocardiographic examination in two-dimensional mode, M mode, pulsed wave (PW) Doppler, continuous wave (CW) Doppler, color flow mapping (CFM), and tissue Doppler. All echocardiographic examinations were performed under isoflurane anesthesia using the same anesthetic protocol for all groups. Anesthetic depth was clinically standardized at the time of image acquisition based on the absence of eyelid and pedal withdrawal reflexes, loss of postural tone, and a stable thoracoabdominal respiratory pattern, with monitoring of respiratory rate/depth and mucosal color throughout the procedure.

The echocardiographic examinations were performed by one experienced examiner using Vivid T9 (GE Healthcare^®^) equipment with an electronic sector scan transducer (12–7.5 MHz). In M-mode, in the right parasternal transverse view at the level of the chordae tendineae, the internal diameters of the left ventricle (LV), end-diastolic (LVEDd) and end-systolic (LVEDs), were measured to calculate the shortening fraction (SF%) using the formula SF% = [(LVEDd-LVEDs)/LVEDd]X 100 [30].

In the apical four-chamber view, the end-diastolic volume (EDV) and end-systolic volume (ESV) of the left ventricle were measured using the modified Simpson method to calculate the ejection fraction (EF%) using the formula: EF% = [(EDV-ESV)/EDV] X 100 [30]. In the same section, the transmitral flow was acquired, and the maximum velocity peaks of the E wave (early ventricular filling) and A wave (late ventricular filling) were measured, and the E/A wave ratio was calculated. The isovolumic relaxation time (IVRT) was obtained in the apical five-chamber view using an intermediate flow between the mitral inflow and the aortic flow, and the E/IVRT ratio was evaluated.

Pulsed tissue Doppler was used to acquire velocity waves derived from myocardial movement, with the Sm wave (systolic displacement of the mitral valve annulus), Em wave (rapid ventricular filling), and Am wave (late ventricular filling) obtained by the four-chamber apical view, with the sample volume positioned in the interventricular septum (septal) and the free wall of the left ventricle near the mitral annulus (parietal). The ratios between the E wave of transmitral flow and the peak parietal tissue velocity Em (E/Em) and Em/Am parietal were calculated.

Because heart rate showed numerical variation among groups during echocardiographic assessment, a sensitivity analysis adjusted for heart rate was performed for tissue Doppler S’ indices. The group-related differences remained after adjustment, indicating that heart rate variation alone did not explain the observed tissue Doppler findings.

### Euthanasia and Sample Collection

At the end of the experimental period, the animals were anesthetized with 100 mg/kg of 10% ketamine (Syntec do Brasil Ltda., Santana de Parnaíba-SP, Brazil), combined with 10 mg/kg of 2% xylazine (Rhobifarma Indústria Farmacêutica Ltda., Hortolândia-SP, Brazil) [31]. A median laparotomy was then performed to collect blood samples from the inferior vena cava using disposable syringes previously heparinized. The heart, lung, and tibia were removed, cardiac samples were weighed, and the length of the tibia was measured. To calculate the cardiac mass index, the ratio between heart mass and tibia length was used, with results expressed in g/mm [32].

### Histopathological Analysis

The removed hearts were washed in cold 0.9% saline solution, dried on filter paper, weighed on a precision scale, and then placed in formaldehyde solution (10%) for 48 h. Subsequently, they were cut transversely, and the steps of dehydration, diaphanization, bathing, and paraffin embedding were followed. The mid-proximal segment was cut into 5 μm sections and stained for collagen with Picrosirius Red. The histological sections of the cuts were analyzed under a Nikon TS 100 optical microscope, and the images were scanned using an optical microcamera system with a 10x objective lens and a final magnification of 100×.

In each session, a photographic record of seven fields of the free wall of the left ventricle was made. Polarized light in the optical microscope was used to identify collagen I and III subtypes, while microphotographs without a polarization filter for total collagen quantification were performed similarly to the protocol used previously [21, 33].

The red staining for Picrosirius Red, indicative of collagen fibers, was separated from the background colors by specific threshold values using the ImageJ 1.6.0_24 image processing program. The manual threshold values were set as Hue (0–10), Saturation (20–255), and Brightness (15–255). The total collagen layer for tissue was defined as the proportion of positive pixels or gray values within the tissue. The ImageJ tool allows the percentage of collagen area deposited in the extracellular matrix to be delimited with respect to the total area of the evaluated field.

### Statistical Analysis

The data were tabulated in Microsoft Excel and analyzed using GraphPad Prism 8.0 (GraphPad Software, San Diego, CA, USA). Data distribution was assessed using the Shapiro–Wilk test. Normally distributed variables were analyzed using parametric methods, whereas non-normally distributed variables were analyzed using nonparametric methods. Descriptive statistics are presented as mean ± standard deviation (SD) or median and interquartile interval/range (IQR), as appropriate.

**Fig. 4:**
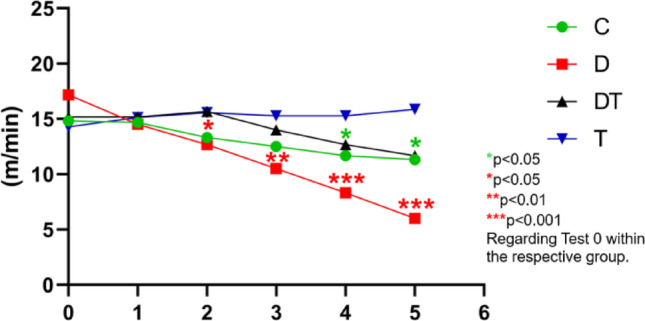
Evolution of exercise tolerance during the experimental protocol. Group C: control (*n* = 6); Group D: doxorubicin (*n* = 6); Group DT: doxorubicin + aerobic training (*n* = 6); Group T: aerobic training (*n* = 7). Values are expressed as mean ± SEM. Longitudinal data were analyzed using a mixed-effects model with restricted maximum likelihood estimation (REML) for repeated measures, followed by Sidak’s post hoc test for multiple comparisons. Statistical annotations indicate significant within-group comparisons relative to Test 0 within respective group. *p < 0.05; **p < 0.01; ***p < 0.001. Non-significant comparisons are not shown

For the analysis of exercise tolerance over time, as show in Fig. 4, longitudinal data were analyzed using a mixed-effects model with restricted maximum likelihood estimation (REML) for repeated measures, accounting for group, time, and group × time interaction effects. When significant effects were identified, post hoc multiple comparisons were performed using Sidak’s test. The Geisser–Greenhouse correction was applied when appropriate. Statistical significance was set at *p* < 0.05. For cross-sectional comparisons among groups at each time point, Kruskal-Wallis tests followed by Dunn’s post hoc tests were used when data were non-normally distributed.

In the final analyses, no observations were excluded on the basis of statistical outlier detection. The analytical dataset for each outcome was defined according to the availability of technically valid measurements for that specific procedure. Therefore, differences in effective sample size across analyses reflect outcome-specific data availability, procedural attrition, or technical loss rather than variable-specific outlier removal. Group allocation was not used as a criterion for inclusion or exclusion. The reduced sample size may have decreased statistical power for some comparisons and should be considered when interpreting the results.

## Results

Statistical differences were found in exercise performance one month after doxorubicin administration and after six months of training. Exercise performance changed differently across groups throughout the experimental period. Group D exhibited a marked decline in performance from Test 0 to Test 5 (65.06%; from 17.5 m/min to 6.5 m/min; *p* < 0.0001). Group C also showed a significant reduction over the same period (23.60%; from 15 m/min to 11 m/min; *p* = 0.0103). In contrast, Group DT showed a smaller numerical reduction in performance (23.07%; from 15 m/min to 11.5 m/min), but this change did not reach statistical significance in the within-group comparison with baseline (*p* = 0.1256). Group T showed a numerical improvement in performance (10.99%; from 14 m/min to 16 m/min), which was also not statistically significant (*p* = 0.2020). The intragroup comparisons are shown in Fig. 4 and in the Supplementary Table S1.

Unless otherwise indicated, the effective sample size for each analysis corresponded to animals with complete and technically valid measurements for the respective outcome; no exclusions were made based on the identification of statistical outliers or group allocation. The evolution of physical performance throughout the experimental protocol was analyzed using a mixed-effects model (REML) for repeated measures. The mixed-effects model showed significant effects of group (*p* < 0.0001), time (*p* < 0.0001), and group × time interaction (*p* < 0.0001), indicating that the response over the experimental period differed between groups (Fig. 4).

Sidak’s multiple comparisons showed a progressive reduction in exercise capacity in Group D, with significant differences from baseline starting at Test 2 and culminating in a 65.06% reduction at Test 5 (*p* < 0.0001). Group C also showed a significant reduction at Test 4 and Test 5, with a final reduction of 23.60% compared with Test 0 (*p* = 0.0103). In contrast, Group DT showed a smaller numerical reduction in performance, reaching 23.07% at Test 5, but this change was not statistically significant compared with Test 0 (*p* = 0.1256). Group T maintained functional capacity during follow-up, with no significant differences compared with Test 0.

No significant intergroup differences were observed between groups at Test 0 and Test 1. From Test 2 onward, significant differences were detected (Test 2: *p* = 0.0013; Test 3: *p* = 0.0009; Test 4: *p* < 0.001; Test 5: *p* = 0.0001; Kruskal–Wallis test with post hoc analysis). At Test 2, Group D showed lower MAS compared with Groups DT and T, whereas Group C also differed from group DT. At Test 3, Group D remained lower than Groups DT and T. At Test 4, group D showed lower MAS compared with Groups DT and T, while Group C also differed from Group T. Finally, at Test 5, Group D was lower than group T, as shown in Table 1.

**Table 1:** Comparison of median (IQR) of maximum aerobic speed (MAS)

	Group C	Group D	Group DT	Group T	*p*-value
	Median (IQR)	Median (IQR)	Median (IQR)	Median (IQR)	
	(*n* = 6)	(*n* = 6)	(*n* = 6)	(*n* = 7)	
Test 0 (m/min)	15 (14.2–16)	17.5 (16.2–18.2)	15 (12.7–17.5)	14 (13–16)	0.0761
Test 1 (m/min)	15 (14–15)	14.5 (13–15.5)	15 (13.7–16.5)	15 (14–16)	0.7693
Test 2 (m/min)	13 (13–14)	12.5^b, c^ (11.5–14.2)	16^a^ (14.7–16.2)	15 (15–16)	0.0013
Test 3 (m/min)	12 (12–13.2)	10.5^b, c^ (9.5–11.5)	13.5 (12.7–16)	15 (14–16)	0.0009
Test 4 (m/min)	12^c^ (11–12)	9^b, c^ (6–10)	12 (12–14)	15 (14–16)	< 0.0001
Test 5 (m/min)	11 (11–12)	6.5^c^ (4.7–7)	11.5 (10.7–13)	16 (15–17)	0.0001

Group C- control (*n* = 6), Group D- doxorubicin (*n* = 6), Group DT- doxorubicin + aerobic training (*n* = 6), Group T- aerobic training (*n* = 7). Superscript letters indicate statistically significant differences between groups at the same time point: a, different from Group C; b, different from Group DT; c, different from Group T. Kruskal-Wallis test followed by Dunn’s post hoc test

Regarding organ weights, a significant overall difference was observed for absolute heart weight (*p* = 0.0029). Post hoc comparisons showed higher absolute heart weight in Groups D and DT compared with Group C. Final body weight differed among groups overall (*p* = 0.0484), with lower values in Group D than in Group T in the post hoc comparision (*p* = 0.0424). The data are presented in Table 2.

**Table 2:** Comparison of median (IQR) values of absolute and indexed heart and lung weights, final body weight, and absolute and indexed lung weights

	Group C (*n* = 6) Median (IQR)	Group D (*n* = 6) Median (IQR)	Group DT (*n* = 6) Median (IQR)	Group T (*n* = 7) Median (IQR)	*p*-value
Heart(g)	1.07 (0.92–1.13)	1.29^a^ (1.22–1.40)	1.28^a^ (1.17–1.34)	1.08 (1.04–1.22)	0.0029
Lung (g)	1.70 (1.65–1.76)	1.81 (1.77–2.01)	1.85 (1.59–1.98)	1.70 (1.63–1.96)	0.3631
BW final (g)	559.6 (552.9–577.1)	513.2^c^ (416.6–543.8)	540.2 (478.1–557.8)	564.1 (554–612.8)	0.0484
Heart/Tibia (g/mm)	0.026 (0.023–0.029)	0.030 (0.029–0.036)	0.031 (0.028–0.032)	0.026 (0,025 − 0,029)	0.0196
Weight lung/BW	0.030 (0.028–0.038)	0.035 (0.032–0.050)	0.036 (0.032–0.038)	0.031 (0.028–0.032)	0.0290

Group C: control (*n* = 6); Group D: doxorubicin (*n* = 6); Group DT: doxorubicin + aerobic training (*n* = 6); Group T: aerobic training (*n* = 7). Superscript letters indicate statistically significant differences between groups at the same time point: a, different from Group C; b, different from Group DT; c, different from Group T. Pairwise comparisons were performed using the Kruskal-Wallis test followed by Dunn’s post hoc test

In the echocardiographic evaluation, group D showed significantly lower values than group C in the variables S Wave par (0.040 vs. 0.065) and S Wave sep (0.04 vs. 0.070), representing reductions of 38.4% and 42.8%, respectively, as detailed in Table 3. Because heart rate differed among groups during echocardiographic assessment, a sensitivity analysis adjusted for heart rate was performed for tissue Doppler S’ indices. The group-related differences remained after adjustment, with similar direction and magnitude, indicating that heart rate variation alone did not explain the observed tissue Doppler findings.

**Table 3:** Comparison of median (IQR) or mean ± SD of echocardiographic variables

	Group C	Group D	Group DT	Group T	*p*-value
	(*n* = 6)	(*n* = 6)	(*n* = 6)	(*n* = 7)	
	Median (IQR)/Mean ± SD	Median (IQR)/Mean ± SD	Median (IQR)/Mean ± SD	Median (IQR)/Mean ± SD	
HR (bpm)	360.5 (160.8–460.8)	397 (383.8–413.3)	361 (337.5–415)	175 (150–186)	0.0642
IVSd (cm)	0.150 (0.100–0.200)	0.150 (0.100–0.200)	0.150 (0.100–0.200)	0.200 (0.100–0.300)	0.8424
IVSs (cm)	0.200 (0.200–0.300)	0.200 (0.175–0.200)	0.200 (0.200–0.225)	0.200 (0.200–0.300)	0.2653
LVEDd(cm)	0.900 (0.675–1.025)	0.850 (0.700–0.925)	0.900 (0.775–0.900)	0.900 (0.800–1.000)	0.7808
LVEDs(cm)	0.583 ± 0.1602	0.583 ± 0.1472	0.533 ± 0.1506	0.587 ± 0.0899	0.8899
LVPWd(cm)	0.200 (0.175–0.400)	0.200 (0.100–0.200)	0.200 (0.200–0.200)	0.200 (0.200–0.200)	0.5102
LVPWs(cm)	0.200 (0.175–0.300)	0.200 (0.175–0.300)	0.200 (0.200–0.225)	0.300 (0.200–0.300)	0.5305
EDV (ml)	1.500 (1.000–2.000)	1.000 (1.000–2.000)	1.000 (1.000–2.000)	1.000 (1.000–2.000)	0.7802
ESV (ml)	0.500 (0.000–1.000)	0.000 (0.000–1.000)	1.000 (0.000–1.000)	0.000 (0.000–1.000)	0.7100
SV (ml)	1.000 (0.750–2.000)	1.000 (0.750–1.000)	1.000 (1.000–1.250)	1.000 (1.000–1.000)	0.5726
EF (%)	66.7 ± 13.76	63.8 ± 17.31	66.7 ± 15.49	68.4 ± 6.87	0.9455
FS (%)	35 (26.25–41.75)	33.5 (24.25–39.5)	27.5 (25.75–45.75)	32 (30–38)	0.8422
RWT	0.382 ± 0.151	0.411 ± 0.107	0.403 ± 0.072	0.367 ± 0.083	0.8738
S Wave par	0.065 (0.057–0.080)	0.040^a^ (0.030–0.055)	0.050 (0.047–0.055)	0.060 (0.050–0.060)	0.0248
S Wave sep	0.066 ± 0.016	0.040^a^ ± 0.010	0.052 ± 0.008	0.060 ± 0.016	0.0389

Group C- control, Group D- doxorubicin, Group DT- doxorubicin + aerobic training, Group T- aerobic training. n-number of animals. ^a^Statistically significant difference compared to group C. *HR *beats per minute (Kruskal-Wallis), *IVSd *interventricular septum in diastole (Kruskal-Wallis), *IVSs *interventricular septum in systole (Kruskal-Wallis), *LVEDd *left ventricular end diastolic diameter (Kruskal-Wallis), *LVEDs *left ventricular end systolic diameter (one-way ANOVA), *LVPWd *left ventricular end diastolic posterior wall (Kruskal-Wallis), *LVPWs *left ventricular end systolic posterior wall (Kruskal-Wallis), *EDV *end-dia-stolic volume (Kruskal-Wallis), *ESV *end-systolic volume (Kruskal-Wallis), *SV *stroke volume (Kruskal-Wallis), *EF* ejection fraction ((one-way ANOVA), *FS *fractional shortening (Kruskal-Wallis), *RWT *relative wall thickness (one-way ANOVA), S Wave par (Kruskal-Wallis), S Wave sep (one-way ANOVA)

An adjusted sensitivity analysis was performed using generalized linear models (GLM/OLS), with the S’ wave as the dependent variable and group and heart rate as covariates. After adjustment, the group effects remained significant, maintaining a direction and magnitude similar to those observed in the unadjusted model. In this adjusted sensitivity analysis, but not necessarily in the unadjusted group comparison shown in Table 3, reductions in S Wave par were observed in the D (β = −0.029; 95% CI − 0.047 to − 0.011) and DT (β = −0.019; 95% CI − 0.036 to − 0.002) groups compared to the C group.

Heart rate showed an independent association with tissue Doppler parameters (parietal S’, *p* = 0.003; septal S’, *p* < 0.001), but did not negate the differences between the groups. Taken together, these findings indicate that, although heart rate may influence tissue Doppler measurements, the differences observed between the groups are not explained exclusively by its variation, reducing the likelihood that anesthesia-related changes are the primary determinant of the results.

**Fig. 5:**
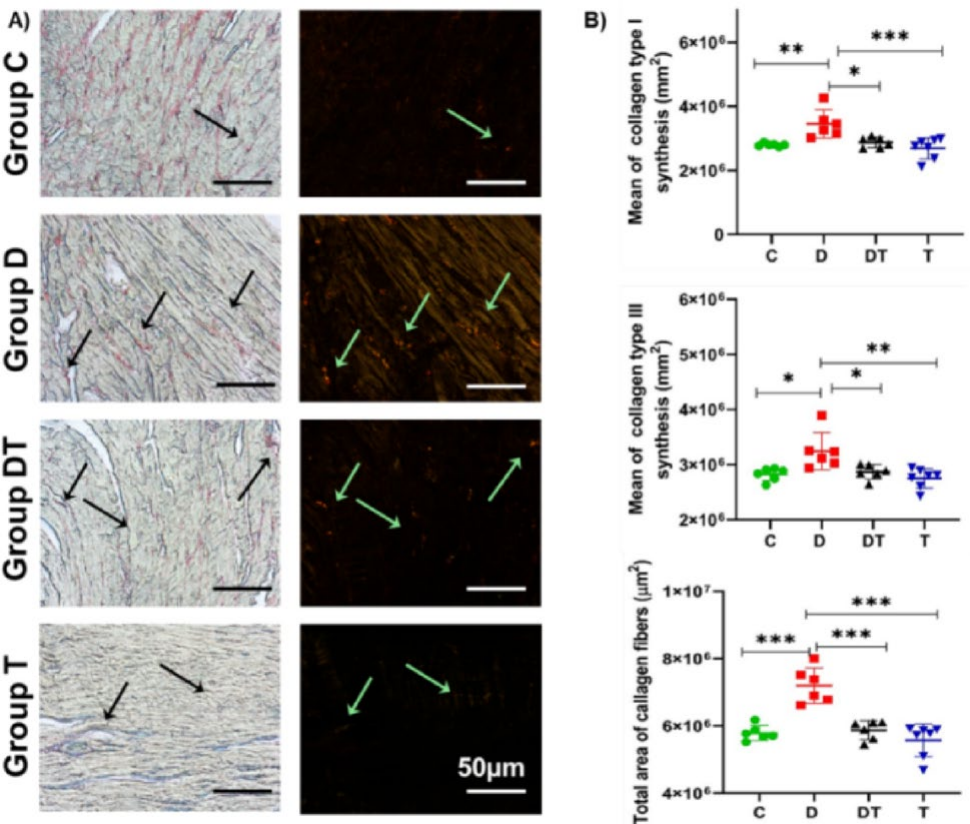
Myocardial collagen deposition assessed by Picrosirius Red staining. **A** Representative photomicrographs of left ventricular tissue sections stained with Picrosirius Red in Group C- control (*n* = 6), Group D- doxorubicin (*n* = 6), Group DT- doxorubicin + aerobic training (*n* = 6), Group T- aerobic training (*n* = 7). Images on the left were obtained without polarized light, showing total collagen deposition. Images on the right were obtained under polarized light, allowing differentiation of collagen subtypes: type I collagen (red/orange fibers) and type III collagen (green fibers). Arrows indicate areas of collagen accumulation. Final magnification: 200×. **B** Quantitative analysis of collagen content expressed as mean ± standard deviation (SD). Group D showed significantly higher levels of type I, type III, and total collagen compared to groups C, DT, and T. Statistical analysis was performed using one-way ANOVA followed by post hoc tests. Significance levels: *p < 0.05, **p < 0.01, ***p < 0.001

In the histological analysis, group D showed higher levels of type I (*p* = 0.0006), type III (*p* = 0.002), and total collagen (*p* < 0.0001) compared to groups C, DT, and T. This confirms the higher deposition of collagen fibers in the myocardium of animals treated exclusively with doxorubicin, as illustrated in (Fig. 5).

## Discussion

The findings observed in the present study are consistent with an HFpEF-like cardiotoxicity phenotype rather than a definitive diagnosis of heart failure, characterized by preserved left ventricular ejection fraction in association with reduced exercise tolerance, cardiac remodeling, increased heart weight, and myocardial fibrosis. However, because invasive hemodynamic assessment, natriuretic peptide measurements, and a more comprehensive evaluation of diastolic function were not performed, these findings should not be interpreted as establishing a definitive diagnosis of HFpEF. Low-intensity aerobic training carried out over 25 weeks, starting 30 days after doxorubicin application, was able to attenuate the increase in myocardial collagen deposition, partially preserve cardiac structure and function, and promote improved tolerance to physical exertion. These findings support the role of exercise as a non-pharmacological strategy for preventing or mitigating the cardiotoxic effects of doxorubicin.

The duration of the aerobic training protocol should also be considered when interpreting the translational relevance of the present findings. Although the 25-week intervention is long relative to the lifespan of rats and does not directly reproduce the absolute duration of most clinical exercise programs, it was intentionally designed to capture the chronic and delayed nature of doxorubicin-induced cardiotoxicity and the long-term physiological adaptations to exercise. Thus, the translational value of this model lies primarily in demonstrating the potential protective effect of sustained low-intensity aerobic training in a chronic cardiotoxic setting, rather than in replicating a specific clinical exercise prescription.

Doxorubicin cardiotoxicity in adult rats is characterized by inflammatory processes in the myocardium, accompanied by intense infiltration of mononuclear cells and collagen deposition [34, 35]. The intensity of the inflammatory response is variable and depends on the cumulative dose of the drug [36]. Several methodologies for inducing cardiotoxicity have already been used and described in the literature, reinforcing the information described above. This experimental model has already been used in our laboratory, and it is known that, at this dose administered to adult Wistar rats, cardiotoxicity has an inflammatory characteristic, with fibrosis resulting from it, and produces echocardiographic changes, which were identified in the present study.

Taken together, the findings are compatible with an HFpEF-like cardiotoxic phenotype, characterized by preserved LVEF in association with reduced exercise tolerance, myocardial fibrosis, and evidence of structural remodeling [37]. However, because invasive hemodynamic assessment, circulating biomarkers, and a more comprehensive diastolic evaluation were not performed, these findings should not be interpreted as establishing a definitive diagnosis of heart failure.

The results obtained in this study using a predominantly aerobic exercise training protocol that began 30 days after the end of doxorubicin treatment support the hypothesis that exercise is capable of at least partially maintaining the physical condition of adult rats. In our study, we observed a drop in tolerance to physical exertion in the group treated with doxorubicin, with a 65.06% reduction in performance (< 0.001).

In contrast, the group treated with doxorubicin and subjected to exercise training showed a smaller numerical reduction in performance of 23.07%, which did not reach statistical significance compared with baseline (*p* = 0.1256). This reduction was closer in magnitude to that observed in the control group, which showed a 23.60% reduction (*p* = 0.0103), and markedly lower than that observed in the doxorubicin-only group. These findings suggest that low-intensity aerobic training may partially attenuate the decline in exercise tolerance induced by doxorubicin [21].

It has already been shown that low to moderate intensity aerobic exercise, started before treatment with doxorubicin, preserves cardiac function, which directly implies a reduction in fatigue on physical exertion, a recurrent symptom in heart failure [21]. Data found in another study show that exercise started after exposure to chemotherapy is important for improving physical condition and functional capacity, mitigating the deleterious effects of chronic non-communicable diseases [20].

The doxorubicin administration model used in our study was effective in inducing cardiotoxicity in the animals, as demonstrated by the increase in absolute heart weight observed in the doxorubicin-treated groups. Cardiotoxicity was also confirmed histologically by the presence of increased collagen. These cardiac alterations may be related to myocardial dysfunction and may resemble features observed in heart failure-like phenotypes; however, the present study was not designed to establish a definitive diagnosis of heart failure, which is a complex syndrome with an uncertain diagnosis in various clinical and experimental settings. Chronic heart failure guidelines classify heart failure phenotypes according to left ventricular ejection fraction, including reduced, mildly reduced, and preserved ejection fraction phenotypes [38]. The findings in the present study are more consistent with an HFpEF-like phenotype.

As emphasized above, cardiac dysfunction resulting from treatment with doxorubicin varies in intensity and may or may not produce a model of HFpEF. In the present experimental model, the findings were compatible with an HFpEF-like phenotype, since no reduction in LVEF was demonstrated. This interpretation is supported by the reduced exercise tolerance and increased heart weight observed in doxorubicin-treated animals, which may reflect myocardial remodeling and early functional impairment. These findings are in line with the literature that uses these parameters as classic markers of cardiac dysfunction [39–41].

Our findings suggest that aerobic exercise may be a useful intervention because, despite the HFpEF-like cardiotoxic phenotype induced by doxorubicin, animals in the doxorubicin plus training group showed better preservation of exercise tolerance than animals treated with doxorubicin alone. This finding supports the potential role of aerobic exercise as a non-pharmacological therapy in doxorubicin-induced cardiomyopathy, bringing clinical benefits in maintaining physical capacity [42–45].

In the present study, it was evident that overall ventricular function was preserved, as assessed by the echocardiographic parameters of left ventricular ejection fraction (LVEF) and shortening fraction, which showed no change. This data reproduced what was found in another physical training model in which swimming was used after treatment with doxorubicin [20].

Although we did not find a reduction in LVEF, tissue Doppler showed significant changes in the S waves (septum/wall) in Group D compared to Group C. Analysis of these waves makes it possible to verify the systolic movement of the mitral annulus and is an important index that can show systolic dysfunction caused by early damage to cardiac tissue. These results suggest that low-intensity aerobic training may attenuate early myocardial dysfunction and left ventricular remodeling associated with doxorubicin-induced cardiotoxicity. This finding has been previously reported in an animal model study with cardiomyopathy of another etiology [15].

An additional point that should be considered when interpreting the echocardiographic findings is the potential influence of isoflurane anesthesia on heart rate and myocardial function. Isoflurane may affect Doppler-derived parameters, including tissue Doppler indices, in a dose-dependent manner. In the present study, the anesthetic protocol and clinical criteria for anesthetic depth were standardized across groups to reduce this source of variability. Moreover, sensitivity analyses adjusted for heart rate showed that the differences in tissue Doppler S’ indices persisted after adjustment, suggesting that heart rate variation alone does not fully explain the observed group differences.

Myocardial collagen plays an essential role as a support structure for the heart’s systolic and diastolic activity. It is directly involved in processes that cause cardiac dysfunction. Under normal conditions, type I collagen accounts for 90% of this extracellular matrix, providing rigidity to the myocardium and transmitting the necessary contraction force during the systolic shortening and twisting of cardiac fibers [46]. In turn, type III collagen makes up around 12% of this structure, forming connections between the type I collagen bundles and acting in the physiological configuration of anatomical structures [46], allowing resistance to pathological deformations and thus ensuring the correct positioning of cardiac structures [47]. However, the exacerbated increase in collagen fibers deposition compromises ventricular compliance, developing pathological hypertrophy, which is characterized by an imbalance between cardiomyocytes and collagen fibers, which impairs ventricular function and structure, leading to cardiac dysfunction [48].

Histological analysis showed greater collagen deposition in the animals treated with doxorubicin alone. In contrast, the groups that underwent training showed less myocardial fibrosis, indicating that exercise also acted positively on cardiac remodeling. This is in line with other studies describing the potential of exercise to prevent chemotherapy-induced fibrosis [49].

Our study showed that group D had a greater amount of Type I, III and, Total collagen fibers than the other groups. The reduction in this response pattern promoted by low-intensity aerobic exercise may be able to attenuate myocardial fibrosis in rats treated with doxorubicin.

The duration of the aerobic training protocol represents a relatively long intervention when considered in relation to the life expectancy of rodents. This design was conceived to allow for the development of chronic cardiotoxic effects and sustained physiological adaptations to exercise. Although the absolute duration does not directly correspond to clinical exercise programs, it can be interpreted proportionally, since longer interventions in animal models are frequently used to approximate chronic exposures in humans. However, our findings should be understood as reflecting the effects of prolonged and continuous exercise, which may not be directly generalized to short-term clinical interventions.

Finally, our findings suggest that animals treated with doxorubicin and submitted to low-intensity aerobic training showed partial preservation of exercise tolerance, lower myocardial fibrosis, and attenuation of structural and functional cardiac alterations.

## Importance and Limitations of the Study

This study has important strengths that support the relevance of its findings. It addresses a clinically meaningful problem in cardio-oncology by investigating whether low-intensity aerobic training, a potentially feasible and accessible non-pharmacological strategy, can mitigate doxorubicin-induced cardiac injury. In addition, the study combined functional assessment, echocardiographic evaluation, and histological analysis of myocardial collagen deposition, providing a more integrated characterization of the cardiac effects of doxorubicin and of the potential protective role of exercise. Another strength is that the training protocol was initiated after doxorubicin exposure, which increases the translational relevance of the model by approximating a rehabilitative rather than purely preventive intervention. Furthermore, the prolonged follow-up allowed the evaluation of chronic adaptations and late manifestations of cardiotoxicity, which are particularly relevant in the context of doxorubicin-related cardiac damage.

At the same time, several limitations should be considered when interpreting these findings. First, the experimental model does not fully reproduce the clinical context of doxorubicin exposure in humans. In clinical practice, doxorubicin is administered to patients with cancer, often in the presence of systemic disease, comorbidities, and concomitant treatments that may influence the development and progression of cardiotoxicity. In contrast, the animals included in the present study were otherwise healthy and did not have cancer or other comorbid conditions. Therefore, although this model is appropriate for investigating mechanisms of doxorubicin-induced cardiac injury and the effects of exercise training, its translational applicability to oncology patients is inherently limited.

Second, echocardiographic assessment was performed under isoflurane anesthesia, which may influence heart rate and myocardial function in a dose-dependent manner and, consequently, affect Doppler-derived parameters, including tissue Doppler indices. Although the anesthetic protocol and the clinical criteria used to define anesthetic depth were standardized across groups, we did not perform objective monitoring of anesthetic depth, such as end-tidal isoflurane measurement, capnography, or individualized MAC-based control. Therefore, residual interindividual variability in anesthetic sensitivity and its potential impact on heart rate and tissue Doppler measurements cannot be completely excluded. Importantly, however, the group differences observed in S’ persisted after adjustment for heart rate in sensitivity analyses, supporting the consistency of the main findings.

Third, although the study demonstrated that low-intensity aerobic training attenuated myocardial fibrosis associated with doxorubicin exposure, the molecular and cellular mechanisms underlying this effect were not investigated. Accordingly, the biological pathways through which exercise modulates collagen deposition and myocardial remodeling remain to be clarified in future mechanistic studies specifically designed for this purpose.

Another limitation is the absence of gold-standard diagnostic measures for a more comprehensive characterization of the heart failure phenotype, such as invasive hemodynamic assessment, circulating biomarkers, and a more detailed evaluation of diastolic function. Therefore, the term HFpEF-like cardiotoxic phenotype is used descriptively and should not be interpreted as a definitive diagnosis of heart failure.

In addition, outcome-specific reductions in sample size may have decreased statistical power and limited the precision of some comparisons. Because the analytical sample for each endpoint depended on the availability of complete and technically valid measurements, the robustness of some results should be interpreted with appropriate caution.

Finally, the exercise intervention was limited to a low-intensity treadmill-running protocol. Although this design was appropriate for evaluating the effects of sustained low-intensity aerobic training in this experimental model, it does not allow conclusions regarding the comparative efficacy of other exercise intensities, modalities, or training regimens. Therefore, the present findings should be interpreted specifically within the context of low-intensity aerobic exercise. Despite these limitations, the study provides relevant experimental evidence that sustained low-intensity aerobic training may attenuate myocardial fibrosis and contribute to the preservation of functional and structural cardiac parameters in rats exposed to doxorubicin.

## Conclusion

This study suggests that low-intensity aerobic training may attenuate adverse cardiac effects associated with doxorubicin exposure in Wistar rats. Animals treated with doxorubicin and submitted to exercise showed partial preservation of physical effort tolerance, lower myocardial collagen deposition, and attenuation of structural and functional alterations. Within this experimental setting, doxorubicin-treated animals exhibited a phenotype compatible with HFpEF-like cardiotoxicity, characterized by preserved left ventricular ejection fraction, reduced exercise tolerance, cardiac remodeling, and myocardial fibrosis. These findings provide preclinical evidence that sustained low-intensity aerobic training may mitigate myocardial remodeling and functional impairment associated with doxorubicin-induced cardiotoxicity. Further studies are needed to determine the underlying mechanisms and to assess the translational relevance of these findings in clinical settings.

## Supplementary Information

Below is the link to the electronic supplementary material.

Supplementary Material 1

## Data Availability

No datasets were generated or analysed during the current study.
