# Supplementary material for: Effects of Low-Intensity Aaerobic Training on Cardiac Dysfunction and Myocardial Fibrosis Induced by Doxorubicin in Wistar Rats
Source: Cardiovasc Toxicol. 2026 Jun 30;26(7):70. doi: 10.1007/s12012-026-10150-7 (PMC13319301; doi:10.1007/s12012-026-10150-7)
Supplement: Supplementary file 1 — Supplementary Material 1 [file 12012_2026_10150_MOESM1_ESM.docx]

Table S1. Longitudinal intragroup comparison of maximum aerobic speed (MAS) during the experimental protocol.

| Group | Test | Δ% | p value |
| --- | --- | --- | --- |
| C | Test 0 vs Test 1 | 1.08 | 0.9997 |
| C | Test 0 vs Test 2 | 10.11 | 0.2965 |
| C | Test 0 vs Test 3 | 15.71 | 0.0730 |
| C | Test 0 vs Test 4 | 21.31 | 0.0170 |
| C | Test 0 vs Test 5 | 23.60 | 0.0103 |
| D | Test 0 vs Test 1 | 15.55 | 0.1296 |
| D | Test 0 vs Test 2 | 26.21 | 0.0109 |
| D | Test 0 vs Test 3 | 38.85 | 0.0005 |
| D | Test 0 vs Test 4 | 51.49 | <0.0001 |
| D | Test 0 vs Test 5 | 65.06 | <0.0001 |
| DT | Test 0 vs Test 1 | 0.00 | >0.9999 |
| DT | Test 0 vs Test 2 | 3.3 | 0.9968 |
| DT | Test 0 vs Test 3 | 7.71 | 0.9272 |
| DT | Test 0 vs Test 4 | 16.48 | 0.3399 |
| DT | Test 0 vs Test 5 | 23.07 | 0.1256 |
| T | Test 0 vs Test 1 | 5.95 | 0.8130 |
| T | Test 0 vs Test 2 | 8.96 | 0.3944 |
| T | Test 0 vs Test 3 | 7.00 | 0.7508 |
| T | Test 0 vs Test 4 | 0.00 | 0.7508 |
| T | Test 0 vs Test 5 | 10.99 | 0.2020 |

*Table 1. Comparison of maximum aerobic speed (MAS) throughout the experimental period. Values ​​are expressed as percentage change (Δ%) relative to baseline (Test 0). Statistical comparisons were performed using a mixed-effects model (REML) for repeated measures, followed by Sidak's post-hoc test for multiple comparisons. p-values ​​refer to comparisons between each assessment time point and the respective baseline measurement (Test 0) within the same group. Group C: control (n = 6); Group D: doxorubicin (n = 6); Group DT: doxorubicin + aerobic training (n = 6); Group T: aerobic training (n = 7). Δ% = percentage change relative to Test 0. Differences were considered statistically significant when p < 0.05.*

Table S2. Pairwise comparisons of maximum aerobic speed (MAS) between experimental groups.

| Groups | C vs. D | C vs. DT | C vs. T | D vs. DT | D vs. T | DT vs. T |
| --- | --- | --- | --- | --- | --- | --- |
|  | p value | p value | p value | p value | p value | p value |
| Test 0 | 0.3399 | >0.9999 | >0.9999 | 0.5047 | 0.0729 | >0.9999 |
| Test 1 | >0.9999 | >0.9999 | >0.9999 | >0.9999 | >0.9999 | >0.9999 |
| Test 2 | >0.9999 | 0.0479 | 0.0500 | 0.0189 | 0.0192 | >0.9999 |
| Test 3 | >0.9999 | >0.9999 | 0.0833 | 0.0425 | 0.0007 | >0.9999 |
| Test 4 | 0.4697 | >0.9999 | 0.0402 | 0.0496 | <0.0001 | 0.4335 |
| Test 5 | 0.2604 | >0.9999 | 0.0820 | 0.1438 | <0.0001 | 0.1587 |

*Group C- control (n=6), Group D- doxorubicin (n=6), Group DT- doxorubicin + aerobic training (n=6), Group T- aerobic training (n=7). Kruskal-Wallis test followed by Dunn’s post hoc test.*

Table S3. Post hoc multiple comparisons of heart and lung weights (absolute and indexed) and final body weight among groups.

| Groups | C vs. D | C vs. DT | C vs. T | D vs. DT | D vs. T | DT vs. T |
| --- | --- | --- | --- | --- | --- | --- |
|  | p value | p value | p value | p value | p value | p value |
| Heart(g) | 0.0133 | 0.0252 | >0.9999 | >0.9999 | 0.1165 | 0.1970 |
| Lung (g) | 0.5063 | >0.9999 | >0.9999 | >0.9999 | >0.9999 | >0.9999 |
| BW final (g) | 0.1670 | 0.7375 | 0.9231 | 0.6715 | 0.0424 | 0.3550 |
| Heart/Tibia (g/mm) | 0.0724 | 0.2258 | >0.9999 | >0.9999 | 0.1201 | 0.3621 |
| Weight lung/BW | 0.1859 | 0.2992 | >0.9999 | >0.9999 | 0.1401 | 0.2340 |

*Group C- control (n=6), Group D- doxorubicin (n=6), Group DT- doxorubicin + aerobic training (n=6), Group T- aerobic training (n=7). Kruskal-Wallis test followed by Dunn’s post hoc test.*

Table S4. Pairwise comparisons of echocardiographic variables between groups.

| Groups | C vs. D | C vs. DT | C vs. T | D vs. DT | D vs. T | DT vs. T |
| --- | --- | --- | --- | --- | --- | --- |
|  | p value | p value | p value | p value | p value | p value |
| HR (bpm) | >0.9999 | >0.9999 | 0.6128 | >0.9999 | 0.054 | 0.5169 |
| IVSd (cm) | >0.9999 | >0.9999 | >0.9999 | >0.9999 | >0.9999 | >0.9999 |
| IVSs (cm) | 0.4388 | >0.9999 | >0.9999 | >0.9999 | 0.5671 | >0.9999 |
| LVEDd(cm) | >0.9999 | >0.9999 | >0.9999 | >0.9999 | >0.9999 | >0.9999 |
| LVEDs(cm) | >0.9999 | 0.9217 | >0.9999 | 0.9217 | >0.9999 | 0.9022 |
| LVPWd(cm) | >0.9999 | >0.9999 | >0.9999 | >0.9999 | >0.9999 | >0.9999 |
| LVPWs(cm) | >0.9999 | >0.9999 | >0.9999 | >0.9999 | >0.9999 | >0.9999 |
| EDV (ml) | >0.9999 | >0.9999 | >0.9999 | >0.9999 | >0.9999 | >0.9999 |
| ESV (ml) | >0.9999 | >0.9999 | >0.9999 | >0.9999 | >0.9999 | >0.9999 |
| SV (ml) | >0.9999 | >0.9999 | >0.9999 | >0.9999 | >0.9999 | >0.9999 |
| EF (%) | 0.9837 | >0.9999 | 0.9955 | 0.9837 | 0.9297 | 0.9955 |
| FS (%) | >0.9999 | >0.9999 | >0.9999 | >0.9999 | >0.9999 | >0.9999 |
| RWT | 0.9616 | 0.9848 | 0.9948 | 0.9991 | 0.8770 | 0.9287 |
| S Wave par | 0.0226 | 0.2000 | >0.9999 | >0.9999 | 0.4751 | >0.9999 |
| S Wave sep | 0.0359 | 0.3978 | 0.8773 | 0.5267 | 0.0965 | 0.7546 |

*Group C- control (n=6), Group D- doxorubicin (n=6), Group DT- doxorubicin + aerobic training (n=6), Group T- aerobic training (n=7). HR=Beats per minute (Kruskal-Wallis); IVSd= Interventricular septum in diastole (Kruskal-Wallis); IVSs= Interventricular septum in systole (Kruskal-Wallis); LVEDd= Left ventricular end diastolic diameter (Kruskal-Wallis); LVEDs= Left ventricular end systolic diameter (one-way ANOVA); LVPWd= Left ventricular end diastolic posterior wall (Kruskal-Wallis); LVPWs= Left ventricular end systolic posterior wall (Kruskal-Wallis); EDV= End-dia-stolic volume (Kruskal-Wallis); ESV= End-systolic volume (Kruskal-Wallis); SV = stroke volume (Kruskal-Wallis); EF = ejection fraction ((one-way ANOVA); FS = Fractional shortening (Kruskal-Wallis); RWT= relative wall thickness (one-way ANOVA ); S Wave par (Kruskal-Wallis); S Wave sep (one-way ANOVA).*

Table S5. Pairwise comparisons of myocardial collagen deposition assessed by Picrosirius Red staining

| Groups | C vs. D | C vs. DT | C vs. T | D vs. DT | D vs. T | DT vs. T |
| --- | --- | --- | --- | --- | --- | --- |
|  | p value | p value | p value | p value | p value | p value |
| Collagen type I | 0.0041 | 0.9685 | 0.8994 | 0.0115 | 0.0006 | 0.6576 |
| Collagen type III | 0.0103 | 0.9805 | 0.9226 | 0.0242 | 0.0018 | 0.7383 |
| Total collagen | <0.0001 | 0.9844 | 0.7672 | <0.0001 | <0.0001 | 0.5503 |

*Group C- control (n=6), Group D- doxorubicin (n=6), Group DT- doxorubicin + aerobic training (n=6), Group T- aerobic training (n=7). One-way ANOVA followed by the appropriate post hoc test.*
